# Targeting of a Fixed Bacterial Immunogen to Fc Receptors Reverses the Anti-Inflammatory Properties of the Gram-Negative Bacterium, *Francisella tularensis*, during the Early Stages of Infection

**DOI:** 10.1371/journal.pone.0129981

**Published:** 2015-06-26

**Authors:** Zulfia Babadjanova, Kari Wiedinger, Edmund J. Gosselin, Constantine Bitsaktsis

**Affiliations:** 1 Department of Biological Sciences, Seton Hall University, South Orange, New Jersey, United States of America; 2 Department of Immunology and Microbial Disease, Albany Medical College, Albany, New York, United States of America; Université Paris Descartes, FRANCE

## Abstract

Production of pro-inflammatory cytokines by innate immune cells at the early stages of bacterial infection is important for host protection against the pathogen. Many intracellular bacteria, including *Francisella tularensis*, the agent of tularemia, utilize the anti-inflammatory cytokine IL-10, to evade the host immune response. It is well established that IL-10 has the ability to inhibit robust antigen presentation by dendritic cells and macrophages, thus suppressing the generation of protective immunity. The pathogenesis of *F*. *tularensis* is not fully understood, and research has failed to develop an effective vaccine to this date. In the current study, we hypothesized that *F*. *tularensis* polarizes antigen presenting cells during the early stages of infection towards an anti-inflammatory status characterized by increased synthesis of IL-10 and decreased production of IL-12p70 and TNF-α in an IFN-ɣ-dependent fashion. In addition, *F*. *tularensis* drives an alternative activation of alveolar macrophages within the first 48 hours post-infection, thus allowing the bacterium to avoid protective immunity. Furthermore, we demonstrate that targeting inactivated *F*. *tularensis* (i*Ft*) to Fcγ receptors (FcɣRs) via intranasal immunization with mAb-i*Ft* complexes, a proven vaccine strategy in our laboratories, reverses the anti-inflammatory effects of the bacterium on macrophages by down-regulating production of IL-10. More specifically, we observed that targeting of i*Ft* to FcγRs enhances the classical activation of macrophages not only within the respiratory mucosa, but also systemically, at the early stages of infection. These results provide important insight for further understanding the protective immune mechanisms generated when targeting immunogens to Fc receptors.

## Introduction

For many intracellular bacteria the induction of a robust innate immune response is a critical factor in host protection and bacterial clearance [1–3]. The induction of innate immunity is triggered upon recognition of bacterial components such as lipopolysaccharides, peptidoglycans, or bacterial DNA by cellular receptors and regulated by a number of cytokines including IL-12, TNF-α and IFN-γ [4, 5].

Interleukin-10, an anti-inflammatory cytokine secreted by many different cell populations including T cells, B cells, macrophages, dendritic cells and keratinocytes [6, 7] inhibits pro-inflammatory cytokine synthesis and the antigen presenting ability of monocytes/macrophages and dendritic cells [8]. A number of studies have demonstrated that many intracellular pathogens, such as *F*. *tularensis*, a Gram-negative intracellular bacterium that causes tularemia, use IL-10 to evade the host immune defense especially in the initial stages of infection [9–12]. *F*. *tularensis* can be transmitted through insect bites, infected carcasses, contaminated water, and inhalation of contaminated air, although inhalation of as little as 1–2 bacteria can lead to respiratory failure and death if left untreated [13]. For this reason, the Centers for Disease Control and Prevention has designated *F*. *tularensis* as a Category A biological agent [14]. Since no licensed vaccine for tularemia is currently available in the United States, there is a need for development of an effective vaccine.

Several studies reported that early innate immune responses, in particular secretion of inflammatory cytokines such as TNF-α, IFN-γ and IL-12, provide immediate control over *F*. *tularensis* replication [15–17]. *F*. *tularensis* infection, however, is characterized by the absence of early immune responses throughout the first 2–3 days after infection [18–20]. This is believed to occur partly because of the low endotoxicity of *F*. *tularensis* lipopolysaccharide (LPS), which is structurally different from other Gram-negative bacterial LPS [21–24]. In addition, despite the absence of an early protective inflammatory response against *F*. *tularensis* infection, the potential role of anti-inflammatory cytokines, such as IL-10, in the progression of infection has not been clearly elucidated.

We have previously demonstrated that targeting inactivated *F*. *tularensis* (i*Ft*) bacteria to the Fcγ receptors (FcγRs) in mice, via immunization with mAb-i*Ft* immune complexes, resulted in: (1) enhanced uptake and presentation of the immunogen (i*Ft*) by professional antigen presenting cells, (2) increased recruitment and activation of dendritic cells in the lungs of immunized mice, (3) enhanced *F*. *tularensis*-specific cytokine and antibody responses, (4) generation of effector memory CD4^+^ T cells, and (5) increased protection against *F*. *tularensis* infection [19, 25, 26]. In the current study we hypothesized that *F*. *tularensis* polarizes antigen presenting cells (APCs) during the first 48 hours post-infection towards an anti-inflammatory status, characterized by IL-10 production, thus allowing the pathogen to avoid protective anti-bacterial innate immune responses. Furthermore, we seek to determine whether targeting of mAb-i*Ft* immune complexes to FcγRs reverses the potential detrimental role of IL-10 during the early stages of *Francisella* infection. Using our vaccine platform we demonstrate that targeting of inactivated *F*. *tularensis* (i*Ft*) bacteria to FcγRs leads to systemic macrophage activation, shifts the cytokine profile from anti-inflammatory to pro-inflammatory, and alters the alveolar macrophage activation state from alternatively to classically activated macrophages in the lungs of mAb-i*Ft* immunized mice during the early stages of *F*. *tularensis* infection. In summary, this study identifies a critical link between the ability of *F*. *tularensis* to suppress the immune response and the ability of FcγR-targeted immunogen to alter that response and thereby enhance protection against infection.

## Materials and Methods

### Mice and bacteria

C57BL/6 and IL-10 genetically deficient mice were purchased from Jackson Laboratories (Bar Harbor, Maine). All mice were housed at the Animal Research Facility at Seton Hall University. The mice were used at 6–10 weeks of age. All protocols were reviewed and approved by the Seton Hall University Ethics Committee utilizing NIH standards.

*F*. *tularensis* LVS (ATCC 29684; American Type Culture Collection) was provided by K. Elkins (U.S. Food and Drug Administration, Bethesda, MD).

### Ethics Statement

All animals were handled in strict accordance with good animal practice as defined by the relevant national and/or local animal welfare bodies, and all animal work was approved by the Seton Hall University Animal Care and Use Committee (Approval # CB1401). Guidelines provided by the NIH were followed in all experimentation. Briefly, mouse anesthesia was performed via i.p. injections of a ketamine / xylazine cocktail, while mouse euthanasia was achieved via CO_2_ administration followed by cervical dislocation.

### Antibodies

Mouse IgG2a anti-*Ft*. LPS mAb used to generate mAb-i*Ft* immune complexes was purchased from Fitzgerald (Cat# 10-F02, clone#M023621, Acton, MA). The following flow cytometry antibodies were purchased from BD Biosciences (San Jose, California): F4/80 (PE), CD11b (FITC), CCR7 (PE-Cy5.5), MHC class II (APC), B7.1 and B7.2 (PercP), CD11c (APC)

### Inactivation and Labeling of *F*. *tularensis*

Inactivated *F*. *tularensis* LVS (i*Ft*) was generated by growing *F*. *tularensis* LVS in Mueller Hinton broth (MHB) media (BD Biosciences) up to a density of 1x10^9^ CFU/mL. The culture was then spun down at 22,000g for 20 min. at 4°C, and washed 3 times with PBS, resuspended in 2% Paraformaldehyde (Sigma) and incubated for 2 hours at room temperature on a rocker. Bacteria were then washed 3 more times with PBS and 1x10^9^ organisms were plated on a chocolate agar plate (BD Biosciences) and incubated for 7 days at 37°C to confirm inactivation. The final concentration of i*Ft* organisms was determined by OD at 610 nm.

### mAb-i*Ft* immune complex (IC) Generation

To generate ICs, 1x10^9^ i*Ft* organisms were incubated at 4°C overnight on a rocker with 0 μg/mL or 1μg/mL of anti-*Ft* mAb in PBS. Following the incubation, i*Ft* or mAb-i*Ft* preparations were administered to mice intranasally. Generation of ICs has been previously confirmed by ELISA and SDS-PAGE [19, 26].

### Immunization and Challenge Studies

C57BL/6 and IL-10 deficient mice were divided into three groups consisting of 5–6 mice/group, 6–10 weeks of age. Each mouse was immunized on days 0 and 21 with 2x10^7^ i*Ft* organisms alone or in the form of mAb plus i*Ft* ICs. On day 35 the mice were challenged with 10,000 CFU of live *F*. *tularensis* LVS. Following challenge survival was monitored twice-daily for 21–25 days. Death due to the infection was considered as the experimental end-point although in the occasions were animals were deemed to suffer (completely immobile, hunched backs, eyes shut), mice were sacrificed via CO_2_ administration followed by cervical dislocation, per our approved animal protocol (Approval # CB1401), and that was considered as the experimental end-point for the particular animals. Exact CFU administered were also verified by culturing and counting the inoculum subsequent to challenge on chocolate agar plate.

### Lung leukocyte isolation

Lungs of immunized mice were harvested 24, 48 and 96 hours post-infection, perfused with cold 1x PBS containing a protease inhibitor cocktail, shredded into small pieces, and placed in digestion buffer containing RPMI (Life technologies), 0.2mg/ml DNaseI (Sigma), 0.4mg/ml Collagenase D (Sigma), and 1M MgCl_2_. After a 30 minute incubation at 37°C the digested tissue samples were forced through a cell strainer and the cell suspension obtained was washed and resuspended in RPMI containing 2% FBS. The cell suspension was then carefully layered on 5 mLs of Lympholyte M (Cedarlane Laboratories- Burlington, NC), and spun down at 15,000g for 30 minutes at room temperature. Following centrifugation, the interface containing the majority of immune cells was obtained and added in RPMI with 2% FBS, prior to enumeration. Identification and enumeration of alveolar macrophages cells was based on the expression of surface antigens F4/80 and CD11b.

### Peritoneal exudate cell (PEC) isolation

PECs were harvested 48 hours post-infection, centrifuged in a refrigerated centrifuge 4,000g for 10 minutes. Following centrifugation, the interface containing PECs was resuspended in RPMI with 10% FBS, prior to enumeration. Identification and enumeration of PECs was based on the expression of surface antigens F4/80.

### Flow cytometry

Peritoneal exudate cells or lung cells were obtained from immunized mice at different time-points post-LVS infection as described above. For cell surface marker staining, cells were washed with PBS-BSA-azide, resuspended in blocking buffer [PBS-BSA-azide plus 30 μg/ml of normal mouse IgG (Sigma)] and incubated on ice for 30 minutes. Cells were then washed with PBS-BSA-azide and fluorescently labeled antibodies to CD11b, F4/80, MHC class II, B7.1, B7.2, CCR7, or their corresponding isotype controls were added. The cells were then incubated on ice for 30 minutes, washed, and then fixed with 2% paraformaldehyde. Cells were then analyzed by flow cytometry on an LSRII flow cytometer (BD Biosciences).

### Cytokine measurements

C57BL/6 mice were immunized intranasally (i.n) with PBS, i*Ft*, or mAb-i*Ft*, boosted on day 21 and challenged on day 35 with 10,000 CFUs of live *Ft* LVS. After 48 hours post-infection peritoneal cells were obtained from all groups and cultured for 24 hours with either LVS (1:10 and 1:100 MOI), *Ft*-LPS (kindly provided by Dr. Timothy Sellati-Albany Medical College, Albany, NY) or *E*. *coli*-LPS (Sigma) at 1 ng/mL, 10 ng/mL and 20 ng/ml, or recombinant IFN-γ (Invitrogen) at 100 U/ml. Supernatants were collected at designated time points and the levels of IL-12p70, TNF-α and IL-10 cytokines were measured using BD Biosciences Cytometric Bead Array (CBA) following vendor instructions.

In a separate experiment, lung tissue (left lobe) was harvested from immunized mice and homogenized (Omni Homogenizer, Omni International). Homogenates were then spun down at 15,000g for 30 minutes at room temperature to remove tissue debris and cytokine levels were detected by using the IL-6, IL-10, TNF-α and IFN-γ ELISA kits by following vendor instructions (Biolegend).

### Statistical Analysis

Statistical differences among the groups were analyzed using a one-way analysis of variances (ANOVA) or the unpaired, one-tailed student t-test. GraphPad Prism 4 provided the software for the statistical analysis (San Diego, CA).

## Results

### Administration of mAb-i*Ft* immune complexes (ICs) reverses the anti-inflammatory properties of LVS *ex vivo* and increases the activation of mouse peritoneal exudate cells (PECs)

**Fig 1:**
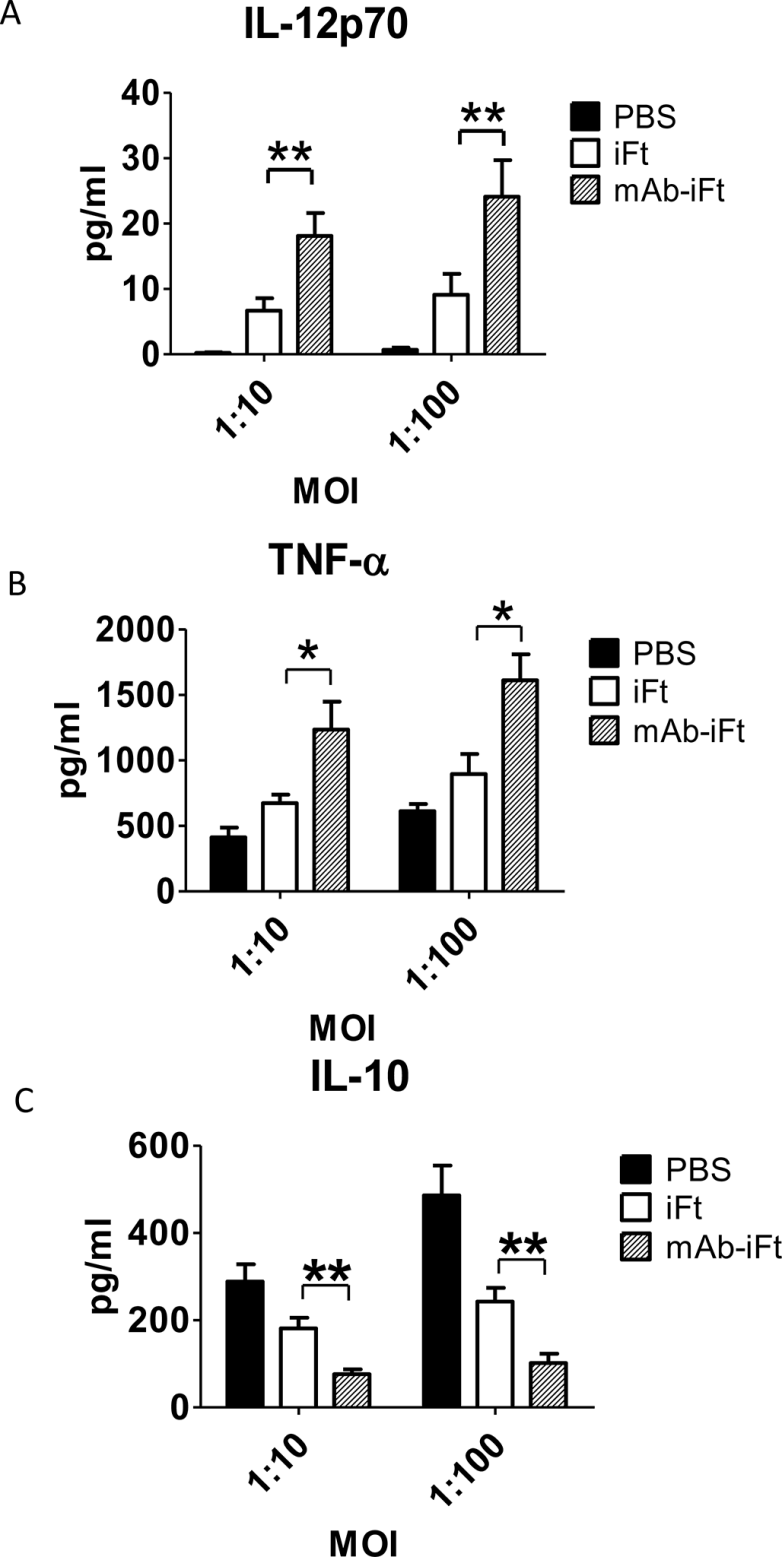
Administration of mAb-*iFt* immune complexes reverses the anti-inflammatory properties of LVS in mouse PECs *ex vivo*. C57BL/6 mice were immunized i.n. with PBS, i*Ft* (2x10^7^ CFUs), or mAb-*iFt*, boosted on day 21 and challenged on day 35 with 10,000 CFUs of *Ft* LVS. After 48 hours post—LVS challenge, the PECs of immunized mice were harvested and cultured in the presence or absence of *Ft*. LVS at 1:10 and 1:100 MOI for 24 hrs. The cytokine production was measured as previously described. Results are representative of three independent experiments. (*) P-value < 0.1; (**) P-value < 0.05; bars represent the SD.

One of the critical immune responses to bacterial infection is the synthesis and release of pro-inflammatory cytokines by innate immune cells during the early stages of infection [1–3]. Given the ability of *F*. *tularensis* to evade the immune system by favoring the short-term secretion of anti-inflammatory cytokines [12, 27], it was of interest to investigate the cytokine levels produced by PECs at the early stages of infection. Therefore, we analyzed the production of inflammatory cytokines IL-12p70 and TNF-α as well as the anti-inflammatory cytokine IL-10 using PECs from immunized and subsequently challenged mice. On day 35 post-immunization mice were challenged with 10,000 CFU of live *F*. *tularensis* LVS and cells were isolated two days post-infection. PECs were further stimulated with LVS *ex vivo* (at 1:10 and 1:100 MOI) for 24 hours and the cytokine levels in the supernatant were measured by the BD Biosciences Cytometric Bead Array (CBA). We observed that the levels of IL-12p70 (Fig 1A) and TNF-α (Fig 1B) in supernatants from PECs of mAb-i*Ft* immunized mice were significantly higher (two to three fold) in comparison to mice immunized with i*Ft* alone. By contrast, the levels of IL-10 production were 2-fold lower in the mAb-i*Ft* compared to mice immunized with i*Ft* alone (Fig 1C) independent of the MOI tested.

IL-10 is known to decrease the cell surface expression of MHC class II and co-stimulatory molecules CD80 and CD86 on murine macrophages [28]. However, one of the mechanisms by which immune complexes trigger immune responses is via T cell activation that requires robust antigen presentation by activated antigen presenting cells and increased expression of co-stimulatory molecules. Previous experiments have shown that co-culturing of *Ft*- specific T cell hybridoma with mouse PECs in the presence of mAb-i*Ft* noticeably increased *Ft*-specific T cell responses compared to using the i*Ft* immunogen alone [26]. In addition, *in vivo* administration of mAb-i*Ft* ICs intranasally increased the activation status of mucosal dendritic cells in the lungs of immunized mice [25]. Therefore, we hypothesized that targeting of immunogen to Fcγ receptors (FcγRs) increases systemically the activation status of antigen presenting cells following LVS challenge. Thus, we examined the expression of MHC class II and co-stimulatory molecules CD80 and CD86 on PECs from immunized mice post-infection.

**Fig 2:**
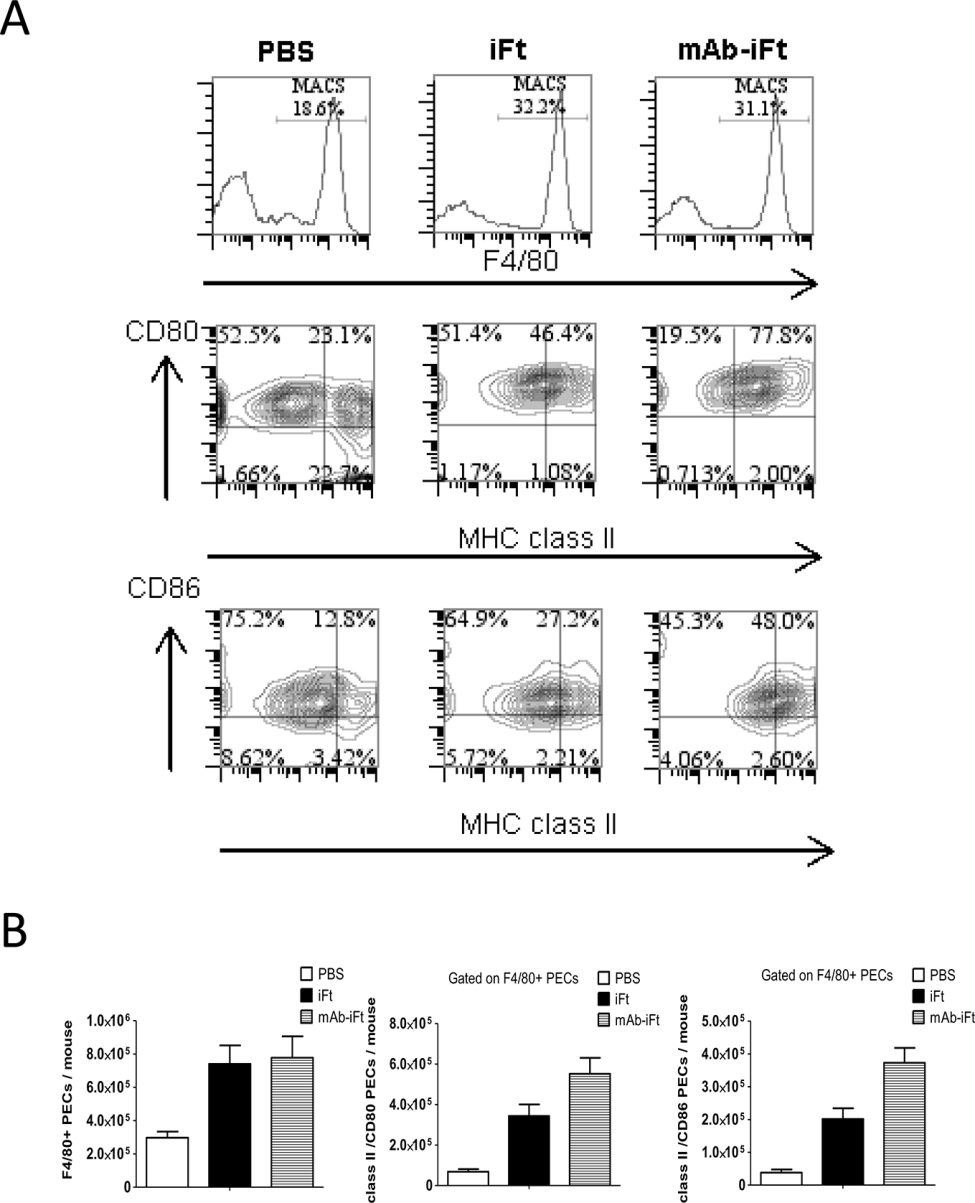
Immunization with mAb-i*Ft* immune complexes increases the activation of PECs following LVS challenge. C57BL/6 mice were immunized i.n. with PBS, i*Ft* (2x10^7^ CFUs), or mAb-i*Ft*, boosted on day 21 and challenged on day 35 with 10,000 CFUs of *Ft* LVS. On day 2 post-infection the peritoneal exudate cells of immunized mice were harvested and the expression of F4/80, MHC class II, B7.1 (CD80), and B7.2 (CD86) were analyzed by flow cytometry. Results are representative of three independent experiments. (*) P-value < 0.1; (**) P-value < 0.05; bars represent the SD.

Peritoneal cells were obtained from immunized mice two days post-LVS challenge as described in Materials and Methods, and the expression of the murine macrophage cell-surface marker F4/80, the co-stimulatory molecules CD80 and CD86, as well as MHC class II was determined by flow cytometry. Although the number of cells expressing the F4/80 cell surface marker was similar between the i*Ft* and mAb-i*Ft* immunized mice, the number and frequency of cells expressing both MHC class II and CD80/CD86 molecules was significantly increased in the mAb-i*Ft* group (Fig 2). This enhancement in surface marker expression upon immunization with mAb-i*Ft* correlates with the increased presentation of i*Ft* in the presence of mAb-i*Ft* in vitro [26].

These results demonstrate for the first time that intranasal immunization with mAb-i*Ft* favors a pro-inflammatory cytokine profile secreted by murine macrophages as depicted by an increase of IL-12p70 and TNF-α production and inhibition of IL-10. Moreover, it triggers activation of peritoneal macrophages during *F*. *tularensis* infection, indicating the induction of a systemic response *in vivo*.

### *F*. *tularensis* LPS contributes to the anti-inflammatory properties of *F*. *tularensis* LVS in an IFN-γ and IL-10 dependent manner

**Fig 3:**
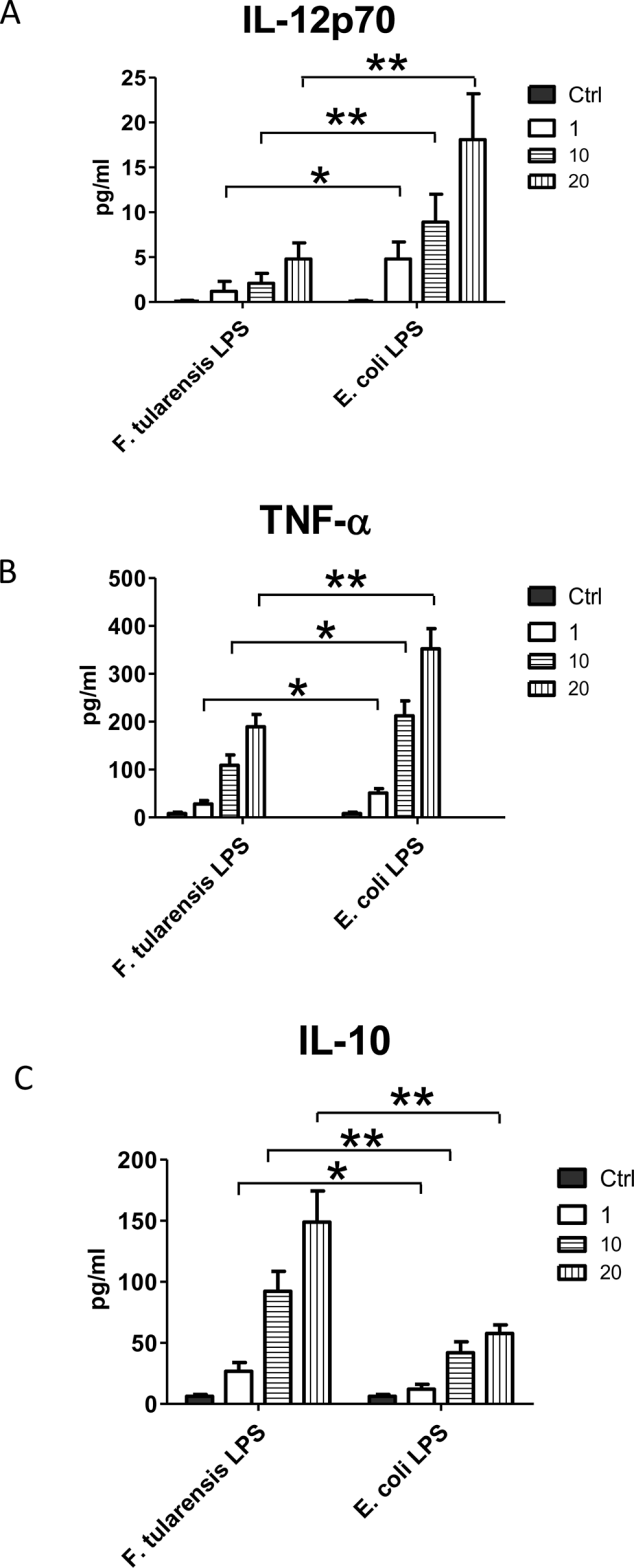
*tularensis* LPS contributes to the anti-inflammatory properties of *F*. *tularensis* LVS during the early stages of infection. ***F*.** PECs from C57BL/6 mice were obtained and cultured in a 96-well plate at 2 x 10^5^ cells/well in the presence or absence of either *Ft*-LPS or *E*. *coli*-LPS at 1 ng/mL, 10 ng/mL and 20 ng/ml. Cells incubated with PBS were used as a control. Levels of IL-12p70, TNF-α and IL-10 were measured using BD Biosciences Cytometric Bead Array (CBA) following vendor instructions. Results are representative of three independent experiments. (*) P-value < 0.1; (**) P-value < 0.05; bars represent SD.

It is well established that *F*. *tularensis* LPS is structurally different from other intracellular bacteria and elicits a subdued inflammatory response in the initial stages of infection, which otherwise are critical in controlling bacterial burden [21–24]. To investigate the effect *F*. *tularensis* LPS on the cytokine profile produced by PECs, we measured the levels of IL-12p70, TNF-α and IL-10 in the supernatants of PECs from naïve C57BL/6 mice cultured with various concentrations of *F*. *tularensis* LPS. As expected, incubation with *F*. *tularensis* LPS at different concentrations triggered significantly lower levels of IL-12p70 (Fig 3A) and TNF-α (Fig 3B) secretion compared to *E*. *coli* LPS (positive control). In contrast, IL-10 production was elevated in the presence of *F*.*tularensis* LPS (Fig 3C). This observation not only confirms the low endotoxic activity of *F*. *tularensis* LPS but also indicates that it down-regulates Th1 cell mediated inflammatory responses via up-regulation of IL-10 and down-regulation of IL-12p70 and TNF-α.

**Fig 4:**
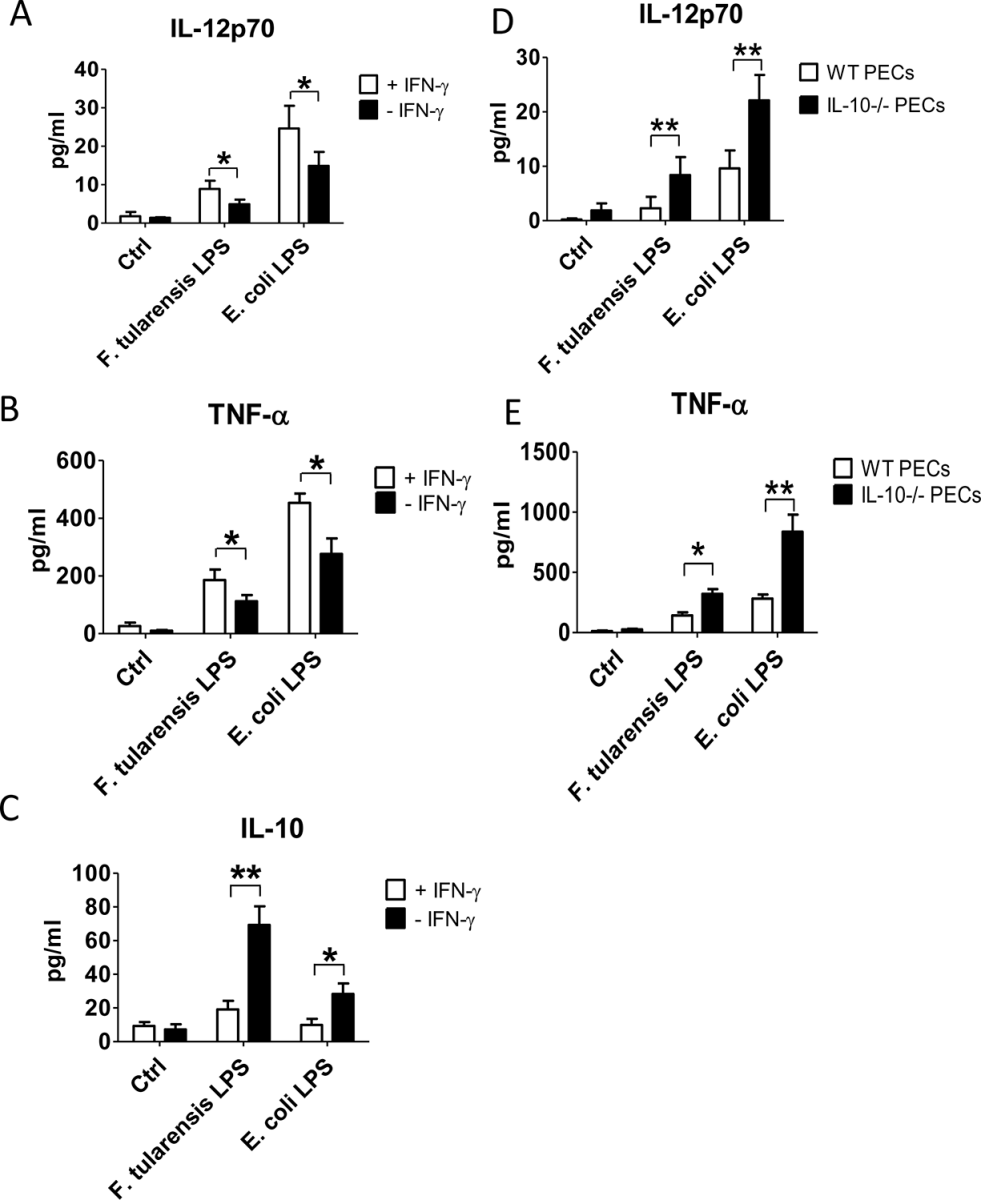
Anti-inflammatory effect of *F*. *tularensis* LPS on mouse PECs is IFN-γ and IL-10 dependent. PECs from naïve and IL-10 genetically deficient C57BL/6 mice were obtained and resuspended in cell culture media. PECs were cultured in a 96-well plate at 2 x 10^5^ cells/well with either *Ft*-LPS or *E*. *coli*-LPS at 1 ng/mL in the presence or absence of recombinant IFN-γ at 100 U/ml. Cells cultured with PBS were used as a control. The cytokine production was measured using BD Biosciences Cytometric Bead Array (CBA) following vendor instructions. Results are representative of three independent experiments. (*) P-value < 0.1, (**) P-value < 0.05, bars represent SD.

Several studies have shown that IL-10 negatively regulates synthesis of IFN-γ as well as synthesis of other pro-inflammatory cytokines in monocytes and macrophages [29–31]. Due to the increased levels of IL-10 synthesis after incubation of PECs with *F*. *tularensis* LPS it was of interest to examine whether this up-regulation correlates with the decrease in TNF-α and IL-12p70 secretion. To investigate this, PECs from C57BL/6 wild-type and IL-10 genetically deficient mice were incubated with *F*. *tularensis* LPS or *E*. *coli* LPS. As previously observed, in the absence of endogenous IFN-γ, *F*. *tularensis* LPS portrayed anti-inflammatory properties via up-regulation of IL-10 production and decrease in the synthesis of IL-12p70 and TNF-α by the PECs. Interestingly, these results were reversed by the addition of exogenous IFN-γ (Fig 4A–4C). Lack of endogenous IL-10, in turn, resulted in an elevated synthesis of IL-12p70 (Fig 4D) and TNF-α (Fig 4E). These results indicate that increased levels of IL-10 synthesis is one of the mechanisms responsible for suppressing the protective pro-inflammatory cytokines such as TNF-α and IL-12p70 leading to an overall suppression of the Th1 response and reduced IFN-γ levels in the early stages of *F*. *tularensis* infection. In fact, the importance of IFN-γ during *F*. *tularensis* infection was reported by Rawool and colleagues [19], who observed a significant drop in survival rate in the mAb-i*Ft* immunized IFN-γ^-/-^ mice compared to the wild type control.

### Administration of mAb-i*Ft* immune complexes reverses the anti-inflammatory properties of LVS in the lungs of immunized mice

**Fig 5:**
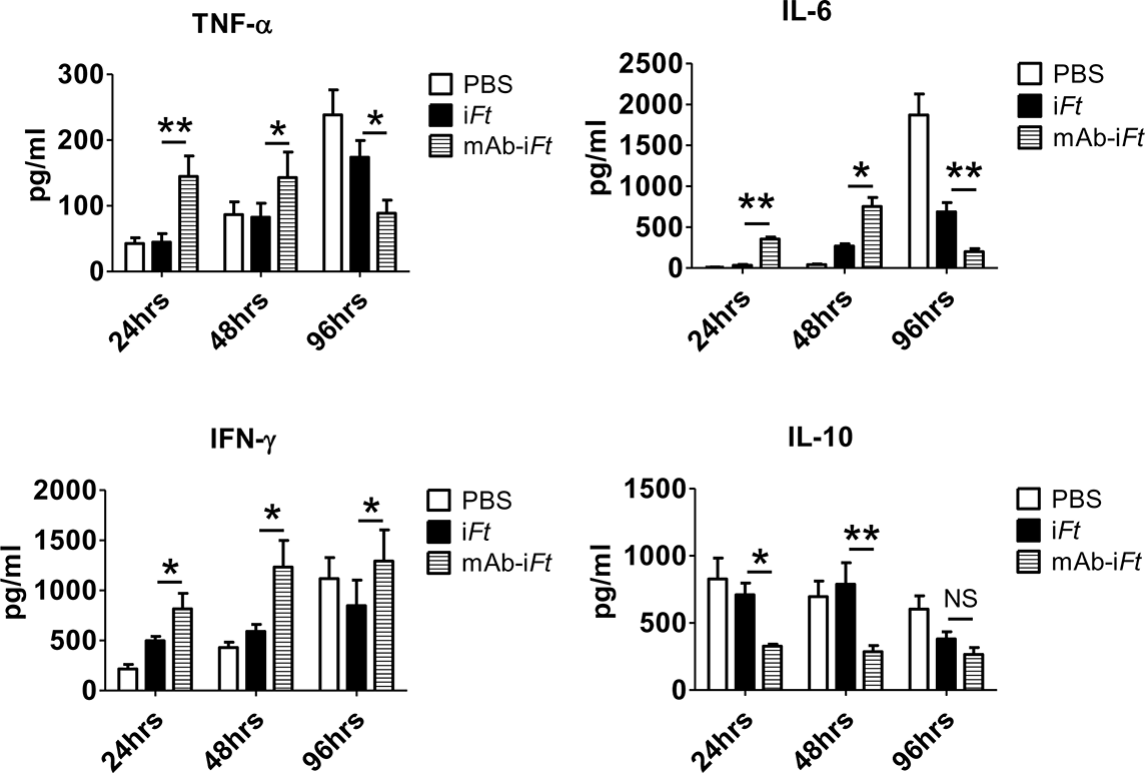
Administration of mAb-i*Ft* immune complexes reverses the anti-inflammatory properties of LVS in the lungs of immunized mice. C57BL/6 mice were immunized i.n. with PBS, i*Ft* (2x10^7^ CFUs), or mAb-i*Ft*, boosted on day 21 and challenged on day 35 with 10,000 CFUs of *Ft* LVS. Lung tissue homogenates were obtained from immunized mice 24, 48 and 96 hours post-infection as indicated above and spun down at 15,000g for 30 minutes at room temperature to remove tissue debris. Cytokine levels were detected by using the IL-6, IL-10, TNF-α and IFN-γ ELISA kits and following vendor instructions (Biolegend). Results are representative of three independent experiments. (*) P-value < 0.1; (**) P-value < 0.05; bars represent the SD.

The balance and kinetics of pro- and anti-inflammatory cytokine secretion during *F*. *tularensis* challenge are key players in controlling the outcome of infection [18, 32, 33]. Consequently, having shown that immunization of mice with mAb-i*Ft* favors a pro-inflammatory cytokine profile secreted by PECs *in vitro*, we attempted to determine the effect of our FcγR targeting approach on the levels of inflammatory cytokines in the lungs of immunized mice during the early stages of LVS infection. To accomplish this, C57BL/6 mice were immunized with PBS, or i*Ft*, or mAb-i*Ft*, boosted on day 21 and infected with a lethal dose of LVS on day 35 post-immunization. The lungs of euthanized mice were harvested after 24, 48 and 96 hours post-challenge, homogenized, and the IL-6, IL-10, TNF-α and IFN-γ cytokine levels were measured by direct sandwich ELISAs. Our results indicate a faster pro-inflammatory response in the lungs of mAb-i*Ft* immunized mice compared to mice immunized with i*Ft* alone, as assessed by the IL-6 and TNF-α production kinetics. Levels of IL-6 and TNF-α peaked at 48 hours post-infection, while they were significantly decreased by day 4 post-challenge (Fig 5). This observation was accompanied by a drop of the bacterial load (data not shown). On the other hand, the pro-inflammatory cytokine levels tested continued to increase at 96 hours post infection in both the PBS and i*Ft* immunized mice accompanied by an increase in the bacterial burden in the lungs (Fig 5 and data not shown). Similar results among the three groups of mice were noted with IFN-γ (Fig 5). On the contrary, the levels of IL-10 were significantly higher in mice immunized with either i*Ft* or PBS versus mAb-i*Ft* within the first 24 of infection, indicating the early anti-inflammatory properties of *F*. *tularensis* LVS. Importantly, the decrease of IL-10 in the mAb-i*Ft* immunized mice observed at 96 hours post-infection was consistent with our previous observation, indicating that FcγR targeting shifts towards a pro-inflammatory cytokine profile at the early stages of *F*. *tularensis* infection (Figs 2 and 5).

### FcγR targeting drives polarization of mouse macrophages towards the AM1 phenotype at the early stages of LVS infection

**Fig 6:**
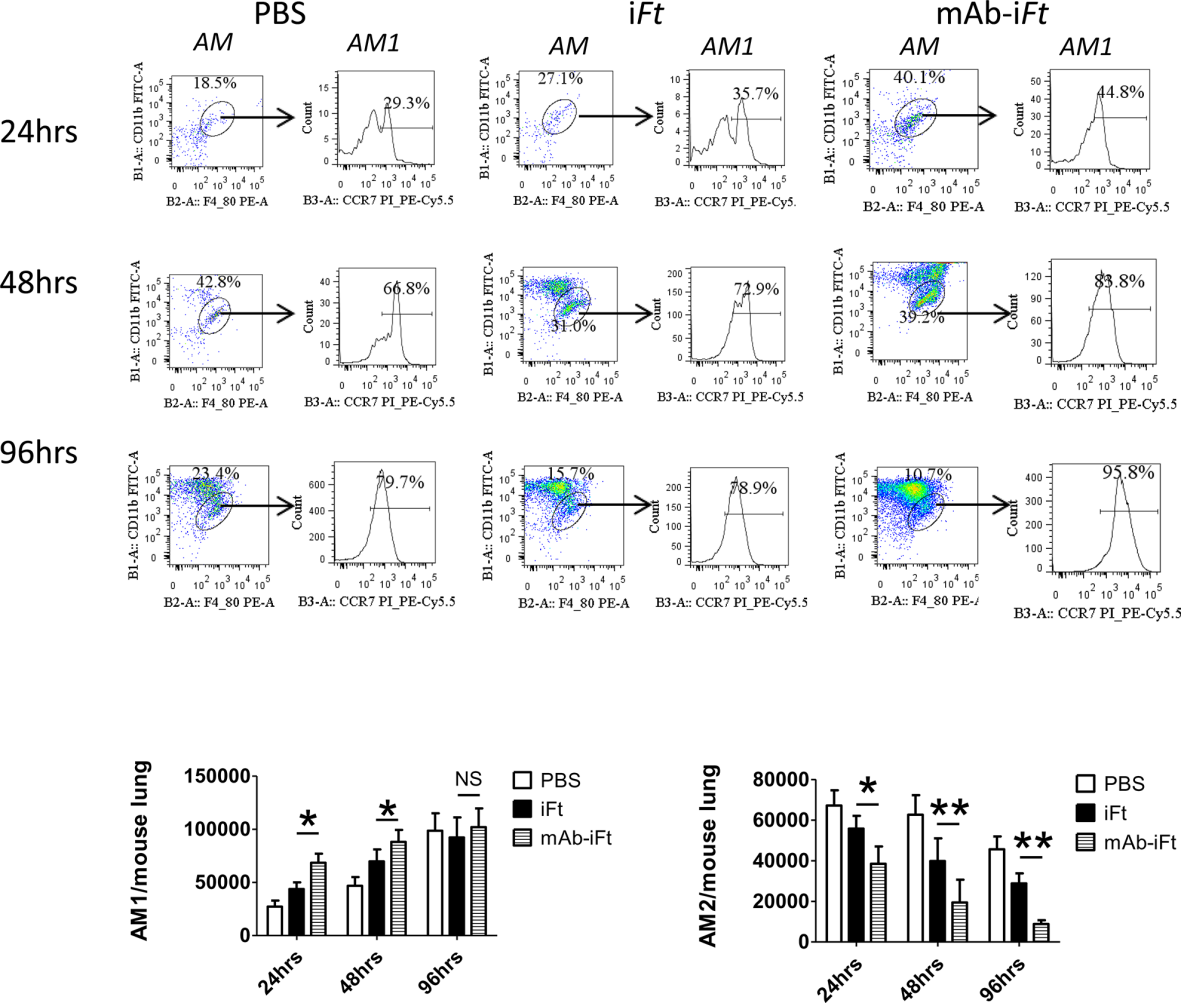
FcγR targeting drives polarization of mouse macrophages towards the AM1 phenotype at the early stages of LVS infection. Lungs of immunized mice were harvested 24, 48 and 96 hours post-infection. For cell surface marker staining, cells were fluorescently labeled with antibodies against CD11b, F4/80, MHC class II, B7.1, B7.2, CCR7, or their corresponding isotype controls were added. Cells were then analyzed by flow cytometry on an LSRII flow cytometer (BD Biosciences). Results are representative of three independent experiments. (*) P-value < 0.1; (**) P-value < 0.05; bars represent the SD.

Our results have suggested that one of the immune evasion mechanisms exploited by *F*. *tularensis* is the reduction of pro-inflammatory cytokines during the early stages of infection. To further investigate possible mechanisms responsible for this shift in the innate immune response, we analyzed the effect of FcγR targeting on macrophage activation and phenotype in the lungs of immunized mice. Previous research showed that one of the ways of *F*. *tularensis* survival and replication within the host cell is its ability to alter the macrophage activation from classically activated alveolar macrophages (AM1) to alternatively activated alveolar macrophages (AM2) [34]. The AM1 macrophages are characterized by high levels of pro-inflammatory cytokines and thus, play an essential role in anti-bacterial innate immune response. In contrast, AM2 macrophages are associated with high levels of anti-inflammatory cytokines, in particular IL-10 [35, 36]. Therefore, we performed flow cytometric analysis to assess the number of AM1 and AM2 present upon immunization with mAb-i*Ft*. C57BL/6 mice were immunized with PBS, i*Ft*, or mAb-i*Ft*, boosted on day 21 and infected with a lethal dose of LVS on day 35 post-immunization. The lungs of euthanized mice were harvested 24, 48 and 96 hours post-challenge and the levels of F4/80, CD11b, CCR7, MHC class II and B7.2 marker expression on white blood cells was analyzed by flow cytometry. Classically activated AM1 cells are characterized as F4/80^+^/CD11b^int^/CCR7^+^/MHC class II^+^/B7.2^+^ cells, while AM2 cells were identified as F4/80^+^/CD11b^int^/CCR7^-^/ MHC class II^-^/B7.2^-^ [35, 37]. Our results indicate that the frequency and number of AM1 cells was significantly higher in mAb-i*Ft* immunized group of mice compared to mice immunized with i*Ft* alone at 24, 48, and 96 hours post challenge (Fig 6). AM that were CCR7+ were also positive for MHC class II and B7.2 and thus considered classically activated macrophages (AM1) while AM that were CCR7- were also negative for the MHC class II and B7.2 markers, indicative of an alternative activated macrophage (AM2) (data not shown). Interestingly, the levels of AM1 cells were comparable among the different immunized groups at 96 hours of post challenge. In addition, our data showed a significantly lower number of AM2 cells in the lungs of mAb-i*Ft* immunized mice relative to mice immunized with i*Ft* alone at all three time points post-infection. It is also of interest that mice immunized with PBS had the highest frequency of AM2 cells, proposing that AM2 polarization *in vivo* may be an additional immune evasion strategy of *F*. *tularensis*, especially at the early stages of infection.

## Discussion

In the current study, we demonstrated that targeting of inactivated *F*. *tularensis* (i*Ft*) bacteria to Fcγ receptors via formation of immune complexes reverses the potential detrimental effect of IL-10 during the early stages of *F*. *tularensis* infection. Early control of bacterial infection depends on the secretion of pro-inflammatory cytokines that regulate the activation of antigen presenting cells and the generation of a protective Th1-like response [1–3]. One of the characteristics of *F*. *tularensis* infection is the lack of such responses during early progression of infection. While some studies depict that this effect is due to the low endotoxicity of *F*. *tularensis* lipopolysaccharide, which is structurally different from other Gram-negative bacteria [21–24], other studies show that the potential up-regulation of anti-inflammatory cytokines is an important factor in the progression of *F*. *tularensis* infection [12, 38]. The latter could possibly be due to suppression of the early activation of inflammasome by *F*. *tularensis*, one of the mechanisms that the bacteria utilizes to evade the early immune response [39–41].

The anti-inflammatory cytokine IL-10 plays a major role in suppression of host immune system by inhibiting the antigen presentation ability of macrophages and dendritic cells mainly by down-regulating the expression of CD80, CD86 and MHC class II molecules, as well as the production of pro-inflammatory cytokines [7]. The ability of IL-10 to suppress innate, inflammatory responses against intracellular pathogens has been previously reported. For instance, lack of endogenous IL-10 production led to a decrease in number of *Trypanosoma cruzi* parasites in the blood of infected mice [9]. Similarly, lack of IL-10 resulted in higher resistance of mice to *Listeria monocytogenes* [42]. In addition, viruses have also evolved mechanisms to escape the immune response by altering the Th1/Th2 balance. Thus, it has been shown that elevated levels of IL-10 correlated with higher viral load in HIV infected individuals [43].

IL-10 has also been identified as a key regulator of immune response to *F*. *tularensis* infection [12]. In particular, IL-10 mediates suppression of IL-17, a cytokine that is essential in the initiation of a protective immune response to *F*. *tularensis* infection [15–17, 44]

It is well established that targeting of antigen to FcγRs using a receptor subclass-specific monoclonal antibodies increases the binding, internalization and processing of an antigen by antigen presenting cells (APCs) in the absence of an adjuvant [45]. Furthermore, targeting of inactivated *F*. *tularensis* bacteria to FcγRs through intranasal immunization of mice with mAb-*iFt* enhances immune response and protection against *F*. *tularensis* infection possibly via FcγR dependent enhanced uptake of the antigen by APCs and further presentation to T cells leading to enhanced T cell activation [19, 26]. In the current study, we showed that a potential mechanism of protection in our *F*. *tularensis* model, following FcγR targeting of fixed bacteria, entails the generation of a Th1 response during the early phases of *F*. *tularensis* infection, by inhibiting the synthesis of the anti-inflammatory cytokine IL-10.

Numerous studies showed that during *F*. *tularensis* infection the early pro-inflammatory responses are significantly suppressed [18, 46], followed by overwhelming up-regulation after 48 hours of infection leading to a severe sepsis [32, 33]. Our results show that i*Ft* targeting to FcγRs up-regulates pro-inflammatory responses at early stages of *F*. *tularensis* infection both *ex vivo* and *in vivo*. Particularly, immunization of C57BL/6 mice with mAb-i*Ft* shifted the cytokine profile towards a pro-inflammatory Th1 type in the lungs and peritoneum of immunized mice during LVS infection, which was associated with reduction in bacterial load. Conversely, immunization with PBS and the inactivated immunogen alone resulted in considerably lower amounts of Th1 cytokines and higher amounts of IL-10 synthesis accompanied by an increase in bacterial burden in the lungs of infected mice. These observations indicate that immunization of animals with mAb-i*Ft* elicits a robust, pro-inflammatory immune response in the early phases of *F*. *tularensis* infection by reversing the inhibitory effect of IL-10 both locally and systemically.

The components of *F*. *tularensis* that contribute to the inhibition of pro-inflammatory responses are not completely understood yet. However, it is well established that *F*.*tularensis* LPS shows low endotoxic activity due to its structural differences from other intracellular pathogens [21–24]. Our findings that *F*. *tularensis* LPS elicits inefficient pro-inflammatory response and up-regulates IL-10 synthesis indicates that LPS is partly responsible for the anti-inflammatory activities of *F*. *tularensis* LVS. The importance of pro-inflammatory cytokines, in particular IFN-γ, in controlling the intracellular infection has been described in previous reports. IFN-γ deficiency in mice during *Listeria monocytogenes* infection compromised activation of macrophages allowing bacteria to escape from phagolysome and further replicate within cytoplasm [42]. Similarly, lack of IFN-γ resulted in decreased survival of mice and increase in bacterial burden during *Legionella pneumophila* infection [47]. In addition, IFN-γ dependent activation of human monocyte-derived macrophages inhibited escape of *F*. *tularensis* into the cytoplasm, thus preventing bacterial replication [48]. In our study we observed that the increased IL-10 synthesis in the early stages of *F*. *tularensis* infection coincided with a decrease of the pro-inflammatory cytokines IL-12p70 and TNF-α in the absence of endogenous IFN-γ. Addition of exogenous IFN-γ to cultures of LPS-treated PECs, however, was able to reverse this suppression. Likewise, an increase in the synthesis of IL-12p70 and TNF-α was observed in IL-10 deficient mice after stimulation with *F*. *tularensis* LPS. Therefore, these data demonstrate that early up-regulation of IL-10 is one of the means of Th1 immune response suppression during early stages of *F*. *tularensis* infection, enabling the bacteria to avoid classical anti-bacterial mechanisms.

The source of pro-inflammatory cytokines during infection is primarily the professional antigen presenting cells, such as dendritic cells and macrophages. Proper activation and differentiation of macrophages is important for the generation of robust, innate immune response against bacterial pathogens. Depending on the extracellular cytokine background, the activated macrophages are divided into two distinct groups, classically activated macrophages (AM1) associated with high levels of pro-inflammatory cytokines and alternatively activated macrophages (AM2) characterized by increased levels of anti-inflammatory cytokines, in particular IL-10 [49]. In fact, IL-10 and CCL17, both produced by AM2, are key players in suppressing induction of AM1 cells [36]. Shirey et al. (2009) demonstrated that *F*. *tularensis* LVS skews macrophage activation from anti-bacterial AM1 phenotype towards AM2, which allows its survival and uncontrolled replication within host cells [34]. Based on these findings, we hypothesized that targeting of mAb-i*Ft* may alter the macrophage phenotype from anti-inflammatory AM2 to pro-inflammatory AM1. Our results showed that immunization of mice with mAb-i*Ft* resulted in higher frequency and number of AM1 cells compared to mice immunized with i*Ft* alone. In addition, our data showed a significantly lower number of AM2 cells in the lungs of mAb-i*Ft* immunized mice relative to mice immunized with i*Ft* alone. Interestingly, immunization with PBS resulted in the highest frequency of AM2 cells, suggesting that AM2 polarization *in vivo* may be an additional immune evasion strategy of *F*. *tularensis*, especially at the early stages of infection.

In summary, we have demonstrated for the first time that targeting of inactivated *F*. *tularensis* bacteria to FcγRs reverses the potential detrimental effect of IL-10 during the early stages of *F*. *tularensis* infection. The anti-inflammatory cytokine IL-10 secreted by *F*. *tularensis* infected murine macrophages promotes the bacterial survival and replication by suppressing the synthesis of pro-inflammatory cytokines TNF-α, IL-12, IL-6 and IFN-γ. The immunization of mice with mAb-i*Ft* triggers activation of macrophages, shifts the cytokine profile from anti-inflammatory towards pro-inflammatory and alters macrophage activation state from alternatively activated to classically activated macrophages. These findings support our hypothesis that targeting bacterial immunogens to FcγRs on antigen presenting cells is an effective way to enhance innate immunity against intracellular pathogens during the early stages of infection. Additional studies using the virulent type A *F*. *tularensis* SchuS4 strain will enhance our understanding of the pathogenesis and evasion mechanisms of *F*. *tularensis*. Further investigation of the IL-10 pathway, as well as identification of additional survival mechanisms utilized by *F*. *tularensis* bacteria will aid the development of new vaccine approaches against *F*. *tularensis* infection.
